# Cardiorespiratory function of patients undergoing surgical correction of Scheuermann's hyperkyphosis

**DOI:** 10.1038/s41598-021-99674-2

**Published:** 2021-10-11

**Authors:** Pablo Vera, Alejandro Lorente, Jesús Burgos, Pablo Palacios, Luis M. Antón-Rodrigálvarez, Rocio Tamariz, Carlos Barrios, Rafael Lorente

**Affiliations:** 1 School of Doctorate, Valencia Catholic University, Valencia, Spain; 2 Institute for Research on Musculoskeletal Disorders, School of Medicine, Valencia Catholic University, Quevedo 2, 46001 Valencia, Spain; 3 Department of Orthopedic Surgery, Hospital Ramón y Cajal, Madrid, Spain; 4 Hospital Virgen del Mar, Madrid, Spain; 5 Servicio de Traumatología y Cirugía Ortopédica, Hospital Universitario HM Sanchinarro, Madrid, Spain; 6 Department of Pediatric Cardiology, Hospital Ramón y Cajal, Madrid, Spain; 7 Department of Orthopedic Surgery and Traumatology, Hospital Infanta Cristina, Badajoz, Spain

**Keywords:** Physiology, Cardiology

## Abstract

The aim of this study was to analyze the impact of surgical correction of the thoracic deformity on the cardiorespiratory function of patients with moderate-severe Scheuermann’s hyperkyphosis (SK). A series of 23 adolescents with SK who underwent surgery through an only posterior approach using all pedicle screw constructs were included in the study. Cardiorespiratory parameters were measured during a maximal exercise tolerance test before and 2 years after surgery. Heart rate, oxygen saturation (SatO2), maximum oxygen uptake (VO2 max), ventilatory capacity at maximal exercise (VEmax), and energy costs were recorded. There were statistically significant differences in the forced vital capacity (FVC) (*P* < 0.05), total VO_2_max (ml/min) (*P* < 0.01), maximum expired volume (VEmax) per minute (*P* < 0.01) and cardiovascular efficiency (HR/VO2 ratio) (*P* < 0.05). None of these changes were clinically relevant. There were no changes in the VO_2_max per kg of body mass. The magnitude of the kyphosis correction did not correlate with the change in normalized VO2max or VEmax. In conclusion, patients with moderate-severe SK improve their baseline respiratory limitations and the tolerance to maximum exercise 2 years after surgery. However, the slight cardiorespiratory functional improvements should not necessarily be attributed to the surgery, and could also be caused solely by the residual growth of the lungs and thorax. Furthermore, respiratory functional changes are under thresholds considered as clinically relevant.

## Introduction

The effects of Scheuermann’s kyphosis (SK) on pulmonary physiology have been poorly studied. Using baseline spirometry, evident ventilatory restrictions were only detected in SK patients with thoracic hyperkyphosis exceeding 100°^1,2^. SK patients with less than 100º hyperkyphosis did not reveal any clinically relevant respiratory limitation. However, a weak but significant correlation between the percentage of predicted FVC and the magnitude of kyphosis was also observed. Clinically, the results found at rest do not constrain functional ventilatory capacity for daily activities for kyphosis less than 80°^1,2^.

Recently, ventilatory functional restrictions were evaluated in patients with Scheuermann’s disease during a maximal exercise tolerance test^3^. Although there were no differences between SK patients and healthy individuals in any of the basal parameters, maximal aerobic power (VO_2_max) and ventilatory capacity (VEmax) were severely deteriorated in SK patients with more than 75° of kyphosis, showing clear respiratory inefficiency. An inverse correlation between the increase in the magnitude of thoracic kyphosis and the deterioration of the maximal aerobic power was also found.

A possible hypothesis is that the functional respiratory limitations described in patients with severe Scheuermann’s hyperkyphosis would benefit from surgical correction of the sagittal deformity. This issue has already been evaluated in patients with severe AIS who showed a limited tolerance to maximal cardiorespiratory exercise before surgery^4^. Both VO_2_max and VEmax remained essentially unchanged 2 years after surgical correction. In that series, most of the AIS patients showed a clear exercise ventilatory limitation, suggesting that, rather than spinal deformity, other factors such as muscle weakness or lack of fitness could be related to the reduced cardiopulmonary function of these patients during maximal exercise. The possible improvement of ventilatory functional restrictions after surgery in patients with severe Scheuermann’s disease has never been analysed.

The objective of this work was to assess the impact of surgery on the cardiorespiratory function of patients with moderate-severe SK (more than 70º). The cardiorespiratory response to incremental exercise was studied in a series of SK patients before and 2 years after surgical correction. Our hypothesis is that similar to AIS patients, surgical correction of the Scheuermann’s thoracic hyperkyphosis by an only posterior approach could improve certain respiratory parameters but without a relevant impact on the cardiorespiratory function of these patients, particularly under demanding circumstances such as intense exercise.

## Methods

### Study design

A prospective study including a consecutive series of patients with SK underwent a maximal cardiopulmonary exercise tolerance test (CPET) to evaluate aerobic capacity and cardiorespiratory function. Patients requiring surgical correction because of the severity of the thoracic deformity (more than 70°), completed a new CPET at the 2-year postoperative follow-up. Only these cases with pre and postoperative CPET were included in the study.

The study protocol was approved by the clinical research ethics committee at the main institution (Ref. #v1:08/05/2016). All patients provided informed consent confirming their agreement to undergo the cardiorespiratory test. In patients below 16 years, an informed consent from the parents was also required. The project follows the regulations of the Helsinki Declaration of 1964 and its subsequent updates.

### Patient inclusion criteria

Patients under the age of 18 years who fulfilled established criteria for SK were recruited from our spine outpatient clinic after a neurologic exam to exclude signs of possible myelopathy related to kyphotic deformity. Patients with pulmonary disorders that could introduce distortions in the results, such as asthma or bronchiectasis, were excluded. According to standardized guidelines, a basal 12-lead electrocardiogram (ECG) was registered in all cases to rule out unknown cardiac dysfunctions preventing maximal exercise^5^.

SK patients performed regular school-prescribed exercise one or two days per week, which is the common amount of exercise at their age in healthy young population. The kyphotic deformity did not prevent SK patients to practice different sports at leisure level. Patients exceeding these limits were excluded from the study since regular athletic training induces better aerobic parameters that could introduce bias into the study. The same inclusion criteria were followed for the CPETs 2 years after surgery. All SK patients were European Caucasian individuals.

### Radiographic assessment

Two spine surgeons, blinded to the results of the CPET, independently measured the magnitude of the total T2-T12 thoracic kyphosis on a full-length lateral radiograph in the normalized standing position. The mean value of the two measurements was used. The two radiographic assessments were made less than four weeks before preoperative CPET and surgery, and less than four weeks before the 2-year follow-up CPET.

### Operative procedure

All patients underwent surgical cor: tion of the deformity by all pedicle screws posterior instrumentation and fusion. Anterior spine release was never used. All surgeries were performed by the same team of spine surgeons (JB and LMAR) following similar surgical principles. The most relevant technical details are briefly described. During posterior exposure, the posterior intervertebral ligamentous structures were carefully preserved particularly at upper and lower spinal segments. Partial facetectomies were performed at all segments aiming at obtaining optimal posterior release for correction. In addition, Smith-Pettersen osteotomies were performed bilaterally in the 3 apical levels of the kyphosis. Using a free-hand technique, bilateral pedicle screws were placed at all levels. The final construct was completed with 6.0-mm precontoured titanium rods correcting the deformity by using cantilever reduction that started from the upper level and with greater compression at apical levels. A mix of autologous bone graft taken from facets, laminae and spinous process and demineralized bone matrix was used for fusion.

As postoperative protocol, none of the patients wore a brace, and started ambulation the second day after surgery. No program of postoperative physical therapy was implemented in any case. Leisure sports activities were allowed from the third month after surgery.

### Cardiopulmonary exercise test

The CPETs were conducted by a specialized cardiologist using a Schiller Cardiovit CS-200 Ergo-Spiro Stress Test System (Baar, Switzerland), which allowed measurement of both static spirometry and cardiorespiratory functional parameters. Baseline pulmonary function was measured on the same day, immediately before the CPET.

The CPETs were conducted under standard conditions of temperature, humidity, and atmospheric pressure^6^. An incremental Bruce exercise protocol for a treadmill was adapted for an ergometer and used following the specifications in a previous study^3^. Briefly, the protocol started with a 5-min warm-up at a speed of 0.75 m/s. Successively, the speed was incrementally increased at 0.2 m/s per minute. The slope of the treadmill was constantly maintained at 1.5%, resembling the normal resistance of air.

Three types of variables reflecting cardiovascular function, metabolic gas exchange, and ventilatory capacity were all measured during the CPET. Cardiovascular function was assessed by recording heart rate (HR) and blood pressure (BP) at baseline, at the aerobic and anaerobic thresholds, and during maximal exercise. Metabolic gas exchange and ventilatory parameters were measured ‘‘breath by breath’’ every 30 s using a respiratory valve and face mask (Hans Rudolph, Inc., Kansas City, MO) with the gas analyser.

Finally, the metabolic equivalents of tasks (METS) were considered to quantify the energy cost that was required for the participants to reach their maximal functional capacity. A MET is defined as the resting metabolic rate, that is, the amount of oxygen consumed at rest, estimated to be approximately 3.5 ml O_2_/kg/min (1.2 kcal/min for a 70-kg person)^7^. As such, work at 10 METS requires ten times the resting metabolism (35.0 ml O_2_/kg/min) and so on. The test duration was also recorded.

### Statistical analysis

The sample size was estimated to detect a difference between two means using VO_2_max as the most pertinent variable. A difference greater than 5 mL/min/kg was considered clinically relevant^8^. To have 80% power to detect a difference, assuming a variance of 32 mL for similar CEPT in our laboratory and a 0.05 two-sided significance level, the minimum required sample size was 20 patients in each group. Statistical analysis was performed using the SPSS 21.0 statistical package (IBM, Chicago, IL). Due to the limited sample size, nonparametric tests were recommended to compare the quantitative variables. Changes after surgery were assessed with the Wilcoxon rank test for paired samples. The probability level (*P*-value) was considered statistically significant for values < 0.05.

### Ethics approval

The study protocol was approved by the clinical research ethics committee at the Hospital Ramón y Cajal, Madrid, Spain (Ref. #v1:08/05/2016).

### Consent to participate

The parents provided informed consent (all patients were less than 18 years old), and patients signed a form confirming their agreement to participate in the study:

Mr./Ms., of years old with ID number, state that he/she has been informed that the project follows the regulations of the Helsinki Declaration of 1964 and its subsequent updates. In addition, I have been informed about the whole project physical health benefits that could be involved in the project “Cardiorespiratory function of patients undergoing surgical correction of Scheuermann's hyperkyphosis” for the personal, social and for research in physiology. Likewise, I have been informed of the type of tests and procedures that will be applied to me and of the objectives of the project, and that I participate non-profit. I have also been informed that my personal data will be protected and included in a file that must be submitted to and with the guarantees of Law 3/2018 of December 5. Taking this into consideration, I GRANT my CONSENT to participate in this investigation

Date:

Signature of the participant:

Signature of the researchers:

### Consent for publication

The signed consent included the agreement to publish the results of the study.

## Results

### Patient’s profile and correction of the deformity

A total of 23 patients with SK that required surgical correction were included in the study. Surgery was indicated because of the severity of the deformity, chronic back pain or aesthetic reasons. Table 1 shows the anthropometric profile of the sample and the characteristics of the thoracic kyphotic curves. Two years after surgery, patients have significantly greater body mass and height, but BMI was roughly unchanged.

**Table 1:** Anthropometric, and thoracic kyphotic curve characteristics of the SK patients before and after surgery.

	Preoperative	2-years Postop	Wilcoxon Rank test	
	Median ± IQR	Median ± IQR	Z	*P*
Age (years)	14 ± 1	16. ± 1	− 4.796	0.000*
Weight (kg)	63 ± 14	65 ± 12	− 3.475	0.001*
Height (cm)	162 ± 5	168 ± 5	− 4.119	0.000*
BMI (kg/m^2^)	23 ± 4.8	23.3 ± 4.7	− 0.243	0.808
T2-T12 Kyphosis (ºCobb)	77 ± 7	45 ± 5	− 4.204	0.000*

IQR: Interquartile range; *P < 0.01.

**Figure 1:**
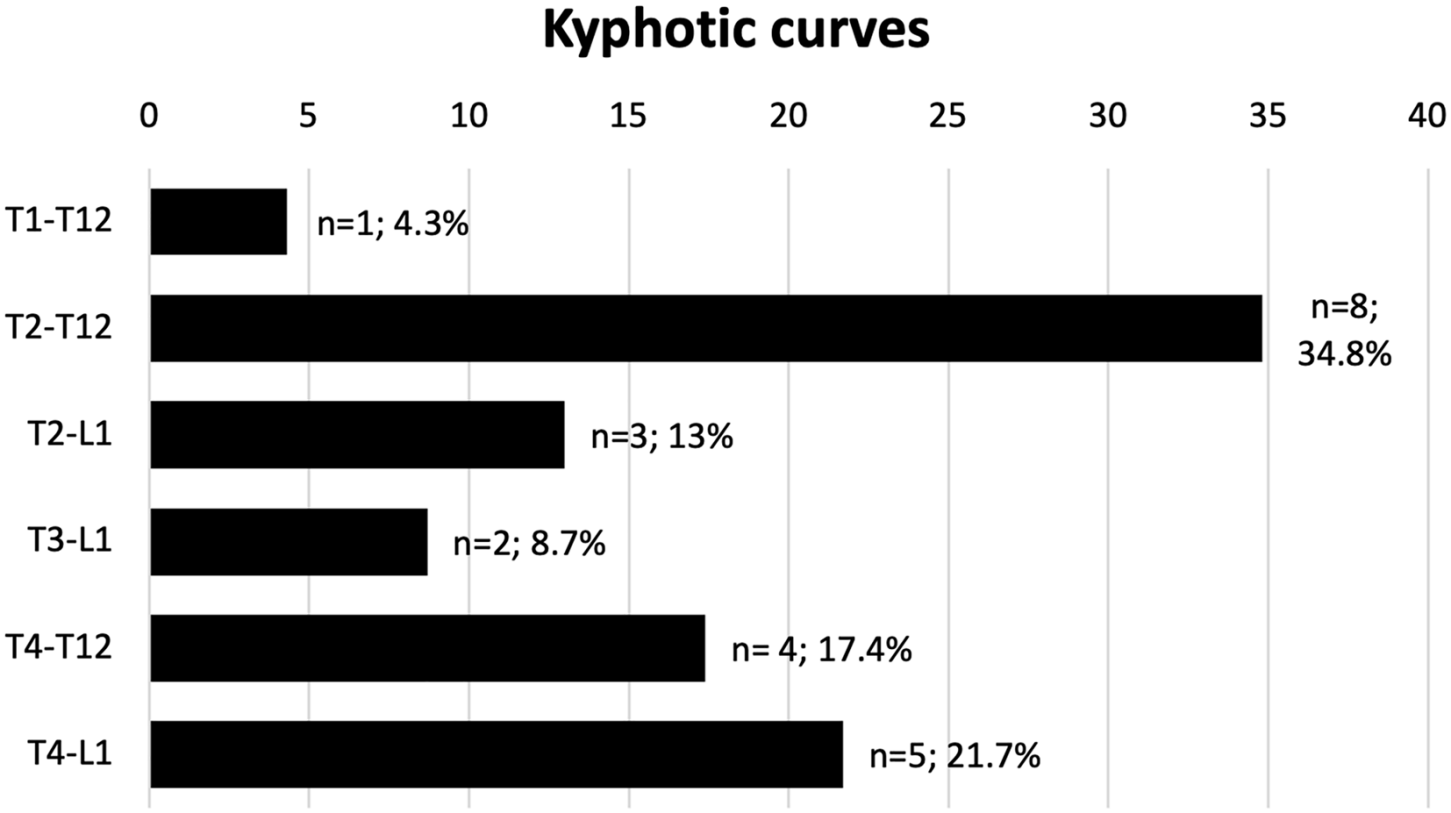
Frequency and involved levels of the kyphotic curves.

The apex of the kyphotic curves was more frequently located at T7 (n = 7, 47.8%) or below (T8: n = 4, 17.4%; T9: n = 4, 17.4%). The most common kyphotic curve involved the T2-T12 levels (Fig. 1). The severity of thoracic kyphosis before surgery was 78.4 ± 5.1° Cobb (minimum: 71º; maximum: 88º). After surgical correction of the deformity, the mean Cobb angle of the thoracic kyphosis was restored to 44.6 ± 2.9° (minimum: 41º; maximum: 50º) (*P* < 0.001). At the 2-year follow-up, the mean correction rate of the T2-T12 kyphotic sagittal angle was 43.0% (95% CI 41.7–44.3).

### Exercise tolerance test before and after SK correction

Table 2 shows the results of the exercise tolerance test according to cardiac, metabolic, and ventilation parameters before and 2 years after surgery. Regarding cardiovascular parameters, the unique significant difference in the surgically treated group between the two exercise tests was found in the systolic blood pressure at maximal exercise being slightly lower after surgery.

**Table 2:** Results of the tolerance exercise test in patients with corrected and uncorrected Scheuermann thoracic hyperkyphosis.

	Exercise Tolerance Test		Before Surgery vs Corrected Wilcoxon Rank test	
	Before Surgery	Corrected Kyphosis		
	Median ± IQR	Median ± IQR	Z	*P*
**Cardiovascular**				
HR basal (bpm)	115.0 ± 24.5	115.0 ± 21.5	− 0.891	0.373
HR max (bpm)	184.5 ± 10.2	184.5 ± 10.2	− 1.598	0.110
Syst. BP (mmHg)	120.0 ± 6.2	120.0 ± 20.0	− 0.973	0.330
Syst. BP max (mmHg)	162.5 ± 30.0	160.0 ± 20.0	− 2.023	0.043*
Sat O_2_ initial (%)	98.0 ± 2.0	98.0 ± 2.2	− 1.204	0.229
Sat O_2_ final (%)	93.0 ± 3.2	93.0 ± 3.0	− 1.685	0.092
**Metabolic**				
VO_2_ max (ml/min)	2.540 ± 126	2.860 ± 125	− 2.618	0.009**
VO2 max/kg	35.0 ± 9.8	35.0 ± 10.0	− 1.141	0.370
VCO_2_ (ml/min)	3340 ± 151	3200 ± 139	− 1.329	0.254
RER (VCO_2_/VO_2_)	1.26 ± 0.15	1.28 ± 0.12	− 1.236	0.216
**Ventilatory**				
FVC	3.8 ± 1.2	4.0 ± 1.0	− 1.978	0.048*
FVC (% of predicted)	67.0 ± 13.5	69.0 ± 13.2	− 3.157	0.002**
VE (L/min)	61.9 ± 30.0	62.5 ± 30.0	− 2.282	0.010*
**Efficiency**				
Respiratory (VE/VCO_2_)	19.6 ± 4.8	20.1 ± 3.3	− 1.034	0.301
Cardiovascular (HR/VO_2_)	81.2 ± 32.6	71.6 ± 36.6	− 2.464	0.014*
Test duration (min)	9.2 ± 1.4	9.3 ± 1.4	− 2.971	0.003*
METS	10.2 ± 2.9	9.9 ± 3.2	− 1.553	0.121

IQR: Interquartile range HR: heart rate; BP: blood pressure; Sat O_2_: saturation O_2_; Pu02: pulse of oxygen; VO2 max: oxygen uptake at maximal exercise; VC02: carbon dioxide output; VE/VCO_2_: expiratory volume and carbon dioxide output ratio; RER: respiratory exchange ratio; VE, ventilation; VE/VO2: ventilatory efficiency; HR/VO_2_: cardiovascular efficiency; mmHg: millimetres of mercury; % of predicted: percentage of predicted FVC according to age, sex, height, mass and ethnicity; L/min: litres per minute); METS: metabolic equivalents of tasks.

*P < 0.05.

**Figure 2:**
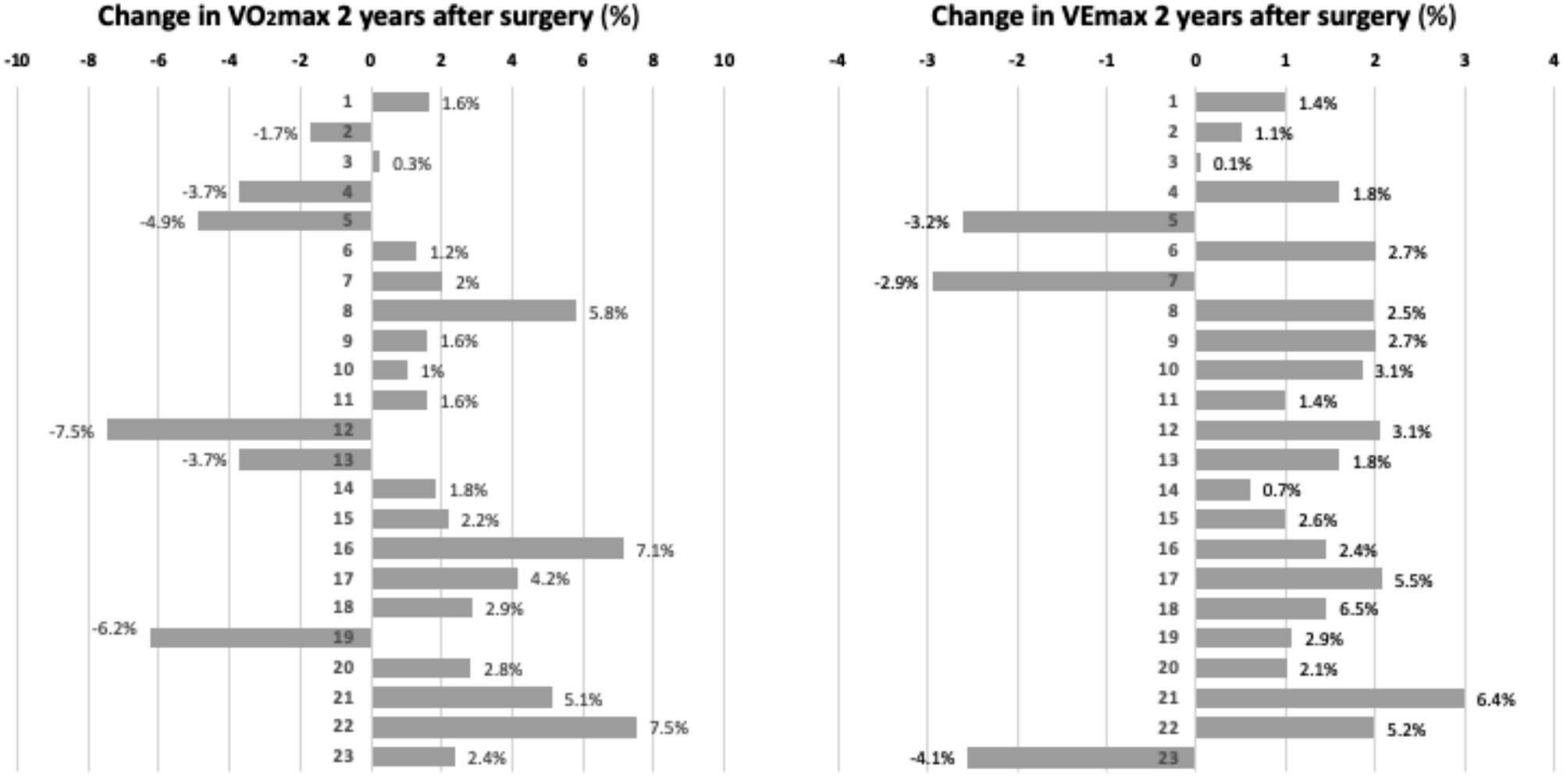
Changes in VO2max (ml/min/kg) and VE (L/min) after surgical correction of the hyperkyphosis as compared to preoperative data.

Without weight normalization, maximal oxygen uptake (VO_2_max ml/min) showed a slight improvement (11.8%) at the 2-year follow-up examination (*P* < 0.01). However, when maximal aerobic power normalized by body weight (VO_2_max in ml/min/kg) was compared, no significant changes were detected before and after surgical correction of SK. The global mean improvement in VO_2_ max was 0.27 ml/kg/min with a maximum of 3.0 ml/kg/min, under the value of 5 ml/Kg/min considered as clinically relevant. Figure 2 shows the 2-year follow-up improvement or decrease of VO_2_max in each patient. Data are given in percentage relative to the preoperative VO_2_max values. In 6 cases (26.1%), the VO_2_max clearly decreased after 2-year follow-up. In 17 cases (73.9%), VO_2_max slightly improved with only three cases surpassing the 5% increase (Fig. 2).

The ventilatory capacity at maximal exercise, measured by VEmax, was relatively low in most cases, both preoperatively and 2 years after surgery. However, VEmax improved slightly after surgical correction of the hyperkyphosis, and the differences were statistically significant (*P* < 0.01) (Table 2). In the first CPET, 8 patients (34.8%) exhibited VE values < 50 L/min, which is considered the lower limit of normal [6, 7]. The global mean improvement in VEmax was 0.92 L/min with a maximum of 2.9 L/min. Figure 2 shows the 2-year follow-up improvement or decrease of VEmax in percentage relative to the preoperative value. Only in 3 cases (13%), the VEmax clearly decreased after 2-year follow-up. In 20 cases (87%), VEmax slightly improved with only three cases surpassing the 5% increase.

**Figure 3:**
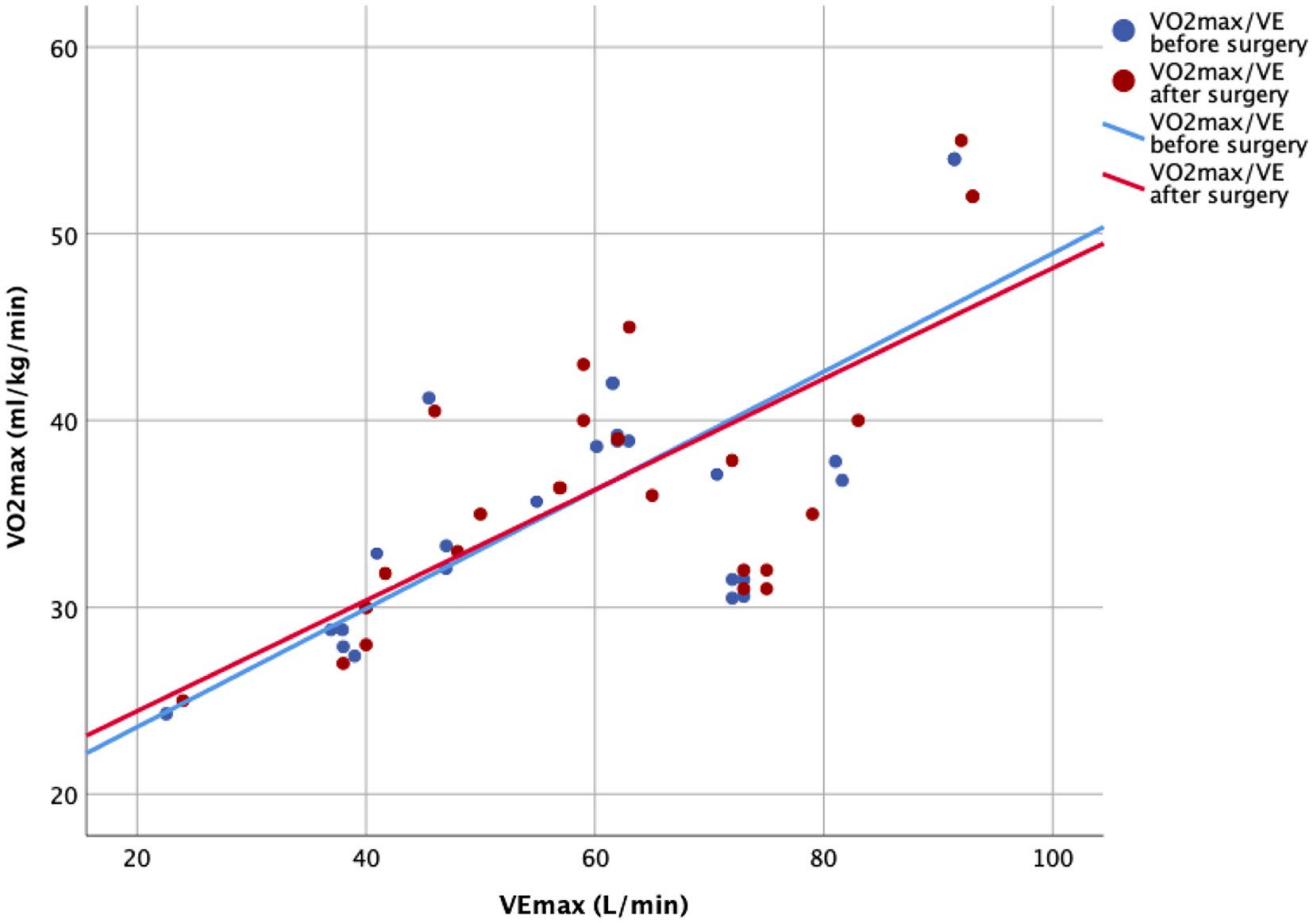
Correlation between VO_2_max y VEmax in the CPET before surgery (r = 0.725; *P* < 0.001) and 2-year after surgery (r = 0.699; *P* < 0.001).

**Figure 4:**
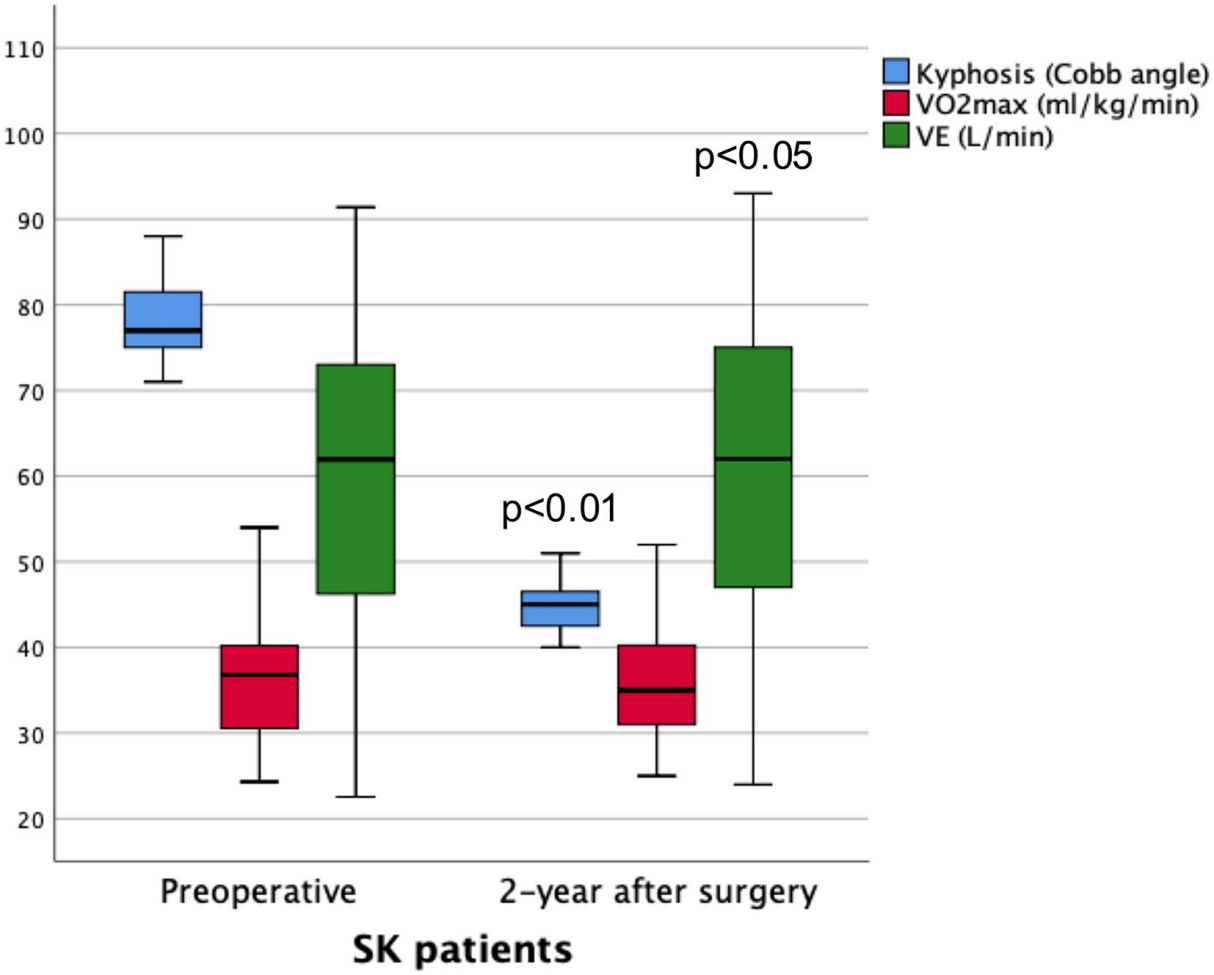
Magnitude of the thoracic kyphotic, maximal aerobic power (VO_2_max in ml/min/kg) and maximal ventilation (VEmax in L/min) in SK patients before and 2-year after surgery.

There was a strong correlation between VO2max and VEmax in both the CPET before surgery and that 2 years after surgery (Fig. 3). Besides the highly satisfactory correction of the kyphosis, VO2max remained almost unchanged after surgery and VEmax showed a small increase but statistically significant (Fig. 4). Cardiovascular efficiency measured by the HR/VO_2_ quotient was still low but also improved significantly after surgery (*P* < 0.05) (Table 2).

The duration of the exercise test was slightly longer after surgery than before surgery (*P* < 0.01). However, there was a small reduction in the energy cost measured in METS (metabolic equivalents of a task) in the test performed after surgical correction of the hyperkyphosis, but without differences in relation to the test before surgery (Table 2).

## Discussion

This study is the first to describe the impact of surgical correction of thoracic hyperkyphosis on the cardiorespiratory functional restrictions consistently found in patients with moderate-severe Scheuermann’s disease. According to previous studies, patients with more than 75º Scheuermann’s hyperkyphosis show clear intolerance to maximal exercise^3^. VO_2_max, VEmax and the HR/VO_2_ quotient (an indicator of cardiovascular efficiency) were severely deteriorated in patients with more than 75° of kyphosis. The current study demonstrates that two years after surgical correction of thoracic kyphosis over 70º in SK patients some respiratory parameters slightly improve: basal FVC, predicted percentage of FVD, total VO_2_max (ml/min) without body mass correction, VEmax and cardiovascular efficiency (HR/VO_2_ ratio). These changes permit surgically corrected SK patients to perform a slightly prolonged maximal exercise test with less hypertensive reaction. However, all these small enhancements of the respiratory parameters were not clinically relevant and cannot be directly related to the surgical correction of the thoracic kyphosis. Interestingly, patients in this series did not change their exercise or sports habits, despite the surgery. None of the patients was more active physically 2 years after surgery.

Analysing pulmonary function with basal spirometry, the occurrence of moderate or severe restrictive lung disease in SK patients was found to be directly related to the magnitude of kyphotic deformity^1,2^. These findings were recently verified by Lorente et al.^3^, who confirmed that both VO_2_max and VEmax values decrease as hyperkyphosis increases in patients with Scheuermann’s disease. This observation reflects the restrictive influence that hyperkyphosis has on ventilatory mechanisms in SK patients. However, in the current study there was no relationship between the magnitude of the sagittal correction of the thoracic spine and the improvement of the respiratory function. In fact, SK patients presented after surgery lower kyphotic magnitudes that the Lorente’s cohort of conservative treated SK cases showing also worse respiratory tolerance to maximal exercise^3^.

Although there is notable variability in treatment decisions among surgeons, operative management of Scheuermann’s hyperkyphosis is often reserved for patients with severe thoracic deformities^9^. Indications for surgery depend on patient age and complaints^10,11^. Functional respiratory restrictions have rarely been claimed as indications for operative treatment. However, adult patients with thoracic hyperkyphosis are prone to experience multiple episodes of acute respiratory failure or chronic respiratory failure requiring prolonged ventilatory support^12,13^. Recently, data regarding respiratory events requiring hospitalization and poor prognosis have been reported in patients with thoracic hyperkyphosis who did not undergo corrective surgery^14^. In this sense, the correction of the spinal deformity could halt the progressive decline of the pulmonary impairment in these cases and might justify the indication of surgery. In addition, if respiratory restrictions increase as hyperkyphosis increases, better ventilatory function could also be expected after surgical correction that might act as a preventive factor for future respiratory adverse events. This issue has never been studied previously.

Similar to that described in AIS patients, the cardiorespiratory limitations at maximal exercise in patients with severe SK remain essentially unchanged 2 years after surgical correction^4^. In the current series, 73.9% of the patients with SK before surgery 65.2% 2 years after surgery had VO_2_max scores below 40–50 mL/kg/min, the expected values for adolescents who are not engaged in regular aerobic training^15^. In 11 patients (47.8%), the VO_2_max values were below 35 mL/kg/minute, indicating an extremely low tolerance to exercise^6,16^. Furthermore, the 8 SK patients (34.8%) that before surgery had VEmax scores under 50 ml, below the expected values for healthy adolescents (range, 50–90 L)^16^, stayed under that figure. These findings indicate no clinically changes in ventilatory limitations during maximal exercise after surgery in SK patients.

In addition, the mean curve correction of the hyperkyphosis was highly satisfactory in the current series (43.0% ± 2.9%), with 33.7° ± 3.8° of kyphosis restoration. There was no correlation between the reduction of kyphosis and the change in either oxygen uptake measured by VO_2_max or VEmax 2 years after surgery. This finding makes unclear the described association between hyperkyphosis and pulmonary impairment in SK patients. It seems that respiratory function is not solely related to the sagittal thoracic profile. At the 2-year follow-up, SK patients who underwent surgery showed significantly less kyphosis, but there were only slight improvements in pulmonary function.

One important point is that the slight cardiorespiratory functional improvement observed in the current sample of SK patients should not necessarily be attributed to the surgical correction of the kyphosis. According to the literature, the normal alveolar development in number is generally complete in number by the age of 2 years^17^. After that age, the alveoli continue to increase in size until thoracic growth is complete at the end of the adolescence period^18,19^. Furthermore, in healthy people, forced expiratory volume in 1 s (FEV1) reaches its maximal level in late adolescence or early adulthood, remaining stable fron the age of 18 to 30 years 20. This finding supports the principle that lung function increases as lungs mature and grow, particularly during puberty^21^. In addition, it has been observed that lung function continues to increase for up to 3 years after body height plateau, indicating that lung growth during puberty is not simply a reflection of body growth^22^. Our patient sample is 14 years of age at the time of surgery (median), did not reached the height plateau and a relevant residual growth was found within the follow-up period of 2 years. With these facts in mind, the slight cardiorespiratory functional improvements detected after surgery might also be caused solely by the residual growth of the lungs and thorax.

In AIS patients, the lack of functional respiratory improvement after surgical correction of the deformity has been explained by generalized skeletal muscle weakness or dysfunction that reduced maximal inspiratory and expiratory pressures during maximal exercise, even in the absence of major ventilatory defects^23^. A similar mechanism would explain the absence of improvements in respiratory function in SK patients. In males with hyperkyphosis, lower spinal muscle density was found as compared to people with less thoracic curvature^24^. It is likely that mechanical limitations on the thoracic cage imposed by hyperkyphosis cannot be compensated for with reduced inspiratory muscle strength, which could induce ventilation restraints during exercise^25^.

In young patients, severe kyphosis may not only distort the lung elastance and global respiratory mechanics which leads directly to restrictive ventilation function but can also affect the development in size of alveoli and capillary vessels, which further affects oxygenation^26^. Scheuermann’s disease commonly begins before puberty and sometimes delays in diagnosis and treatment since very often the deformity is wrongly attributed to poor postural attitudes. The Scheuermann’s kyphotic deformity starting at early adolescence surely cannot interfere with the initial and complete development of alveoli in number but might prevent the alveoli normal growth in size during puberty and late adolescence period. This hypothesis could explain by itself the small changes in the respiratory function between the two CPETs performed with a median age of 14 and 16 years. Evidently, if the thoracic deformity starts at late adolescence after the lungs are almost fully developed, that is not the case in our sample, will not have a significant effect on lung function unless there is an extreme deformity or an associated neuromuscular condition causing respiratory muscle weakening.

This study has some limitations that should be addressed. First, the current research includes a limited number of SK patients who underwent posterior surgery because of moderate-severe hyperkyphosis (> 75°). However, currently, the indication for surgical correction in SK patients is relatively infrequent, and no author has focused on the impact of surgery on respiratory function in these patients. A second limitation is the difficulty in measuring pulmonary function accurately during a maximal exercise tolerance test. The motivation to perform the pre and postoperative CPET could not be the same having a different impact on the outcomes. This limitation is inherent to all studies measuring oxygen uptake during maximal exercise. A third limitation is that respiratory and diaphragmatic muscle function was not analysed. However, the functional evaluation of all these muscles requires at least semi-invasive technology.

In summary, patients with severe SK before surgery show a limited tolerance to maximal cardiorespiratory exercise that slightly improved 2 years after surgical correction of the kyphotic deformity. Despite the surgical restoration of physiologic thoracic kyphosis, SK patients still showed reduced aerobic power, ventilatory restrictions and a respiratory inefficiency. However, two years after surgery, SK patients showed some improvements in their baseline respiratory limitations (FVC and predicted FVC percentage) with limited influence on their tolerance to maximal exercise. The results of the current study suggest that the reduced respiratory function during maximal exercise in SK patients cannot be strictly associated with the magnitude of the kyphotic deformity and could be related to other factors, such as muscle weakness or structural spine stiffness that reduces inspiratory and expiratory thoracic spine movements. Taking into account the age of the patients at surgery, the slight cardiorespiratory functional improvements detected might also be caused solely by the residual growth of the lungs and thorax. Finally, the current findings suggest that a clinically relevant improvement of the pulmonary function or aerobic capacity, providing higher tolerance to maximal exercise, cannot be expected after posterior only surgical correction in SK patients with more than 70º deformity.

## Data Availability

The dataset analyzed during the current study is available from the corresponding author on reasonable request.

## Code availability

The code is available from the corresponding author on reasonable request.
